# Impact of medical specialists' locus of control on communication skills in oncological interviews

**DOI:** 10.1038/sj.bjc.6600797

**Published:** 2003-02-18

**Authors:** Y Libert, P Janne, D Razavi, I Merckaert, P Scalliet, N Delvaux, A-M Etienne, S Conradt, J Klastersky, J Boniver, C Reynaert

**Affiliations:** 1 Université Catholique de Louvain, Faculté de Psychologie et des Sciences de l'Education, Louvain-La-Neuve, Belgium; 2 Université Libre de Bruxelles, Faculté des Sciences Psychologiques et de l'Éducation, Brussels, Belgium; 3 Université de Liège, Faculté de Psychologie, Liège, Belgium

**Keywords:** physicians' locus of control, communication skills, oncological interviews

## Abstract

Although is it widely recognised that physicians' characteristics could influence their communication styles, no empirical evidence is currently available. No studies are available on the impact of physicians' locus of control (LOC) on their communication skills. LOC is a generalised belief regarding the extent to which life outcomes are controlled by an individual's actions (internal LOC) or by external forces such as luck, fate or other individuals (external LOC). It was hypothesised that physicians with external LOC would take more into account others' concerns than physicians with internal LOC and would consequently use more appropriate assessment, informative and supportive functions. A total of 81 medical specialists were assessed in a simulated interview and a clinical interview. Communication skills were rated according to the Cancer Research Campaign Workshop Evaluation Manual. LOC was assessed using the Rotter I-E scale. Communication skills of the upper and lower quartiles of physicians in respect of their scores on this scale were compared using Student's *t*-test. Results show that physicians with external LOC give more appropriate information than physicians with internal LOC in simulated interviews (*P*=0.011) and less premature information than physicians with internal LOC in clinical interviews (*P*=0.015). This result provides evidence that physicians' LOC can influence their communication styles in oncological interviews and in particular the way they provide information to the patient.

Most physicians are aware that communication skills are of great importance and would like to be trained (Calman and Donaldson, 1991). In oncology, the influence of those skills on professional quality of life has been frequently emphasised. For example, in a sample of 393 consultant nonsurgical oncologists in the UK, Ramirez *et al* (1995) found that physicians who felt insufficiently trained in communication and management skills had significantly higher levels of burnout than those who felt sufficiently trained. Researches on physicians' professional quality of life should thus imply the study of determinants of communication skills.

Researches on physicians' professional competence should also imply the study of those determinants. Effective communication skills are, indeed, the key to achieve the three main purposes of physician–patient relationship. They are the necessary tools required to assess (Maguire, 1990), to inform (Fallowfield and Jenkins, 1999) and to support (Novack, 1987) patients adequately. Effective assessment skills promote the expression of cancer patients' concerns (Maguire *et al*, 1996). Information and support giving are effective only if given after exploring patients' feelings and if the information and the support given are realistic and take into account the interview coherence. Unfortunately, using effective skills is particularly difficult when the task is breaking news, the emotional level high and the information complex, as it is the case in oncological interviews.

Although it is widely recognised that physicians' characteristics could influence their communication skills in this context and lead to different communication styles, no empirical evidence is currently available. In their theoretical model, Parle *et al* (1997) underlined the role that outcome expectancy beliefs could play among the determinants of assessment skills promoting the expression of cancer patients' concerns. For those authors, only health professionals who have enough positive outcome expectancies about the consequences of those skills would have the willingness to use them and would consequently give more appropriate information and support to patients.

In psychology and other social sciences, it has been demonstrated for many years that individuals could greatly differ on the personal control that they perceive on their outcome expectancies. This difference has been approached through one of the most studied psychological concept: the locus of control (LOC). LOC was introduced by Rotter (1966) and refers to a generalised belief regarding the extent to which life outcomes are controlled by an individual's actions (internal control) or by external forces such as luck, fate or other individuals (external control). In Rotter's social learning theory, LOC is a personal characteristic that defines the person's position on a continuum between the belief that life outcomes are exclusively controlled by his own actions and the opposite belief that life outcomes are exclusively controlled by external forces. Numerous psychological researches have proven that behaviours of people with internal and external LOC could be greatly different (see for a review Rotter, 1990). To our knowledge, however, LOC concept has never been studied in any empirical study on physicians' communication skills in oncology.

This study aims to make up for this lack of knowledge by exploring the relation between medical specialists' LOC and communication skills used in oncological interviews. Our purpose is to assess whether significant differences exist in communication skills used by physicians with a more internal or external LOC orientation in this context. Our hypothesis proposes that physicians with external LOC, as they believe that life outcomes are controlled by external forces such as other individuals, would take more into account others' concerns than physicians with internal LOC and would consequently use a more appropriate assessment, informative and supportive skills with cancer patients.

Those hypotheses were tested in the analysis of a highly emotional simulated interview and a clinical oncological interview that were performed in the baseline assessment of a randomised communication skills training programme designed for medical specialists dealing with cancer patients. In this analysis, physicians' personal factors previously reported as potentially influent on their communication skills were taken into account (socioprofessional data, previous experiences in communication skills training, attitudes on psychosocial aspects of cancer, level of burnout and level of job stress). Moreover, interviews' and patients' characteristics potentially influent on physicians' and/or patients' behaviours were controlled in the analysis of clinical interviews.

## SUBJECTS AND METHODS

### Recruitment

Data come from the baseline assessment of a randomised controlled communication skills training programme designed for medical specialists and developed in Belgium from 1999 to 2001. All French-speaking Belgian physicians dealing with cancer patients were invited by mail to take part in the training programme (*n*=3706), and all institutions devoted to cancer care were contacted and asked to deliver an internal mail (*n*=2741).

To be included in the study, physicians had to be specialised in medical or surgical oncology, radiotherapy, haematology, gynecology, etc. They had to be working with cancer patients (part time or full time), to show an interest for a psychological training focusing on physician–patient–relative communication and to be willing to participate in the training programme and its assessment procedure. They had also to speak French. Physicians refusing the assessment procedure and those already participating to another psychological training programme during the assessment period were excluded from the study.

### Assessment procedure

Before training, the assessment procedure included two simulated and two clinical interviews (one implying the presence of a relative and the other not) as well as a set of questionnaires. Only results concerning individual simulated and clinical interviews will be reported here. The local ethics committee approved of the study.

### The simulated interview

The simulated interview was audiotaped and videotaped. It implied breaking a breast cancer diagnosis. The actress was trained to maintain carefully the same behaviours and the same high emotional level all over the study. During the interview, her main concern was linked to the disease of her daughter (asthma) that made her less receptive to other personal problems, even to the consequences of her cancer diagnosis. The main characteristic of her role was thus to discard personal implications of the diagnosis except for the consequences of the news on her daughter's well-being. This was carried out to increase the emotional level of the interview and to reduce the possible bias that may derive from the wide range of physicians' specialities. Moreover, the use of real-life highly emotional context for assessment procedures is ethically questionable. A simulated interview was performed for those reasons and because it has been proven to be a valid method to represent how a physician would perform with real patients (Gordon *et al*, 1988) and allows standardising the patient history and reactions (Maguire *et al*, 1996).

Before the interview, the physician had enough time to fill in questionnaires and to read the clinical description and the goals of the interview. He or she was then introduced in the recording room with the actress and told that, after 20 min, the interview would be put to an end. A clock helped time management and the recording room was made to look as realistic as possible.

### The clinical interview

A clinical interview was also audiotaped. Patients were chosen by physicians according to the following inclusion criteria: breaking a news whether bad, neutral or good, patient being more than 18 years old, able to speak and read French, being free of any cognitive dysfunction and having given his written informed consent. Data were provided by physicians following the interview regarding patient's diagnosis (type of cancer, months since diagnosis, disease status), prognosis and current cancer treatment, the type of information (diagnosis related or not) and news (bad, good or neutral).

### Interview rating system

Audiotaped interviews were transcribed by trained secretaries and corrected by psychologists to be rated with an interaction process analysis system (the French translation by Razavi *et al* (1993) of Booth and Maguire's (1991) Cancer Research Campaign Workshop Evaluation Manual (CRCWEM)). The CRCWEM provides a rating of form, function, control, psycho-logical depth, contents and blocking behaviour of each utterance of an interview. We focused our analyses on the functions of the utterances only.

Functions could aim to introduce or close the interview; to assess (elicit, clarify, check or summarise psychosocial or general) concerns; to acknowledge patients' utterances; to give appropriate information; to reassure or express empathy; to interpret the patients' thoughts or beliefs by making educated guesses, confronting patients' thoughts and beliefs or alerting to the reality of the situation; and finally to negotiate which steps have to be taken next. Providing information and reassurance before exploring patients' feelings, of an unrealistic kind or without taking the interview coherence into account, are considered as premature.

Raters were 14 psychologists. They were involved in the whole study and blind to the pre- or post-training status of the interview recordings and never rated any tape that they previously corrected. All raters were intensively trained. The raters' training included: getting information about the rating system, reading the manual, doing rating exercises and being supervised. Every rater had to succeed a validating test before being allowed to analyse the interviews. Before beginning to rate any study tape, every rater had to reach the following concordance criteria with the validating test: 85% for the rating of the forms of the utterances, 67% for the functions, 83% for the blocking behaviours, 71% for the psychological depth of the exchanges and 60% for the content categories. Raters who did not succeed the validating test were further trained and were invited to take part in another validating test. Moreover, all raters were supervised regularly all along the rating process by the raters' coordinator.

### Questionnaires

Before interviews, physicians and patients were asked to complete a set of questionnaires. Before the simulated interview, physicians completed a socioprofessional questionnaire, the Rotter I-E scale, the semantic differential attitude questionnaire (SDAQ), the Maslach burnout inventory (MBI) and the job stress survey (JSS). Before the clinical interview, patients completed a sociodemographic questionnaire, the hospital anxiety and depression scale (HADS) and the multidimensional health locus of control scale (MHLC). Moreover, the evaluator assessed patient's functional impairment using the Karnofsky performance status (KPS).

#### Physicians' socioprofessional data

Data were collected about physicians' age, gender, medical speciality, medical specialisation achieved or not, number of years of practice in medicine and in oncology, number of cancer patients cared in the last week, their type of medical practice and whether or not they had some previous communication skills training in the last year.

#### Rotter I-E scale

The Salehi's (1981) validated French translation of Rotter's I-E scale (Rotter, 1966) was used in this study to measure physicians' LOC. This scale is a 29-item self-report scale with a scoring range from 0 (internal LOC) to 23 (external LOC) excluding six buffer items. It is designed to measure the respondent's perceived ability to influence events in his or her own life. Persons with internal LOC believe that fate and fortune are within their own personal control. In contrast, persons with external LOC believe that external forces such as luck, fate, or other individuals control their lives. To our knowledge, no previous study has assessed the general LOC of medical specialists dealing with cancer patients. Among the few studies in which physicians' LOC has been assessed, only the Revicki and May's (1985) study used the Rotter I-E scale. In their sample of 210 family physicians, they found a mean score of 7.1 (s.d.=4.5).

#### Semantic differential attitude questionnaire (SDAQ)

The French translation (Razavi *et al*, 1993) of the SDAQ (Silberfarb and Levine, 1980) was used to assess physicians' attitudes on the psychosocial aspects of cancer. This questionnaire includes a list of 20 attitudes. The contrasting adjectives remain the same for each concept scored. Each attitude is scored on 13 semantic differential scales ranging from 1 to 7, from the positive to the negative pole. A score of 4, neutral, is allotted whenever an answer is missing. Attitudes are measured by adding up scores obtained for each question on the 13 scales, and dividing the result by 13. The 20 indices are grouped in five factors and a total score. Factors reflect attitudes about oneself (four items), toward cancer and death (three items), personal growth (three items), professional relationships (four items) and occupational attitudes (six items). For each of the five factors, an average index is obtained by averaging the scores of the corresponding factor's constituent attitudes. Total score is obtained by averaging the scores of all attitudes.

#### Maslach burnout inventory (MBI)

The French-translated version (Dion and Réjean, 1994) of the MBI (Maslach and Jackson, 1986) was used to assess physicians' level of burnout. This self-report inventory is a 22-item seven-point Likert scale ranging from never (0) to daily (6). It assesses three dimensions of the burnout syndrome: emotional exhaustion (feelings of being emotionally overextended and exhausted by work) (nine items), depersonalisation (an unfeeling and impersonal response towards patients) (five items) and personal accomplishment (feelings of competence and successful achievement in work with patients) (eight items). In a sample of nurses and physicians (*n*=123) (Dion and Réjean, 1994), a score of 18 or less for emotional exhaustion, 5 or less for depersonalisation and 40 or more for personal accomplishment defines a low level of burnout. By contrast, a score of 27 or more for emotional exhaustion, 10 or more for depersonalisation and 33 or less for personal accomplishment defines a high level of burnout. A middle level is defined by the values included between those scores.

#### Job stress survey (JSS)

We used the French translation of the JSS (Vagg and Spielberger, 1999), a 30-item psychometric instrument designed to assess the perceived severity (intensity) and frequency of occurrence of working conditions that are likely to affect adversely the psychological well-being of employees who are exposed to them. Subjects first rate, on a nine-point scale, the relative amount (severity) of stress that they perceive to be associated with each of the 30 JSS job stressors as compared to a standard stressor event, ‘Assignment of disagreeable duties’, which was assigned a value of ‘5’. Respondents are asked to report, on a scale from 0 to 9+ days, the number of days on which each workplace stressor was experienced during the preceding 6 months. Summing the ratings of each of the 30 individual JSS items provides overall severity and frequency scores as well as an overall job stress index score, based on the sum of the cross-products of the severity and frequency scores. Severity scores from 142 to 150, frequency scores from 99 to 116 and job stress index scores from 59 to 64 indicate an average (median) level of perceived stress severity, frequency and job stress index.

#### Patients' sociodemographic data

Each patient provided demographic information including age, gender and school level completed.

#### Hospital anxiety and depression scale (HADS)

The HADS (Zigmond and Snaith, 1983) is a four-point 14-item self-report instrument assessing anxiety and depression in physically ill subjects. This scale was translated into French and validated in a sample of cancer in-patients (Razavi *et al*, 1990). The use of the total score is recommended to assess psychological distress. Scores from 0 to 12 indicate no disorder, 13 to 18 adjustment disorders and more than 18 major depressive disorders (Razavi *et al*, 1990).

#### Multidimensional health locus of control (MHLC)

The MHLC scale (Wallston and Wallston, 1982) contains 18 items self-report statements concerning beliefs about what controls health. Each statement is ranged on a six-point scale as to the degree of agreement. Three measures are given: health LOC internality (IHLC) (the degree to which an individual believes that luck, chance, fate, or uncontrollable factors are responsible for health or illness), health LOC chance externality (CHLC) (the degree to which an individual believes that luck, chance, fate, or uncontrollable factors are responsible for health or illness) and health LOC powerful others' externality (PHLC) (an individual's beliefs that his or her health or illness is determined by important figures such as physicians and other health professionals or parents). Each HLC subscale scores from 6 (low) to 36 (high).

#### Karnofsky performance status (KPS)

The KPS (Karnofsky and Burcheval, 1949) is a commonly used measure of cancer patient's functional impairment that has adequate inter-rater reliability, concurrent validity and discriminant validity (Grieco and Lung, 1984). A patient scoring under 80 on this scale is not able to achieve daily life activities.

### Statistical analysis

For feasibility reasons, physicians unable to accrue a patient for the clinical interview scheduled in the assessment procedure but who participated in the simulated interview could be considered evaluable. Data were analysed using SPSS10.0 (1999, Chicago, IL, USA). Be owing to the normal physicians' LOC distribution in our sample (Kolmogorov–Smirnov test is 0.089 (*P*=0.171)), parametric statistical analyses were performed. Descriptive analyses were first performed on the total sample. Physicians' and patients' continuous variables of the upper (22%) and lower quartiles (27%) (the cutoff point could not be strictly established to the top and bottom 25% of the Rotter I-E scale distribution because of *ex aequo* scores) of physicians in respect of their Rotter I-E scale scores were compared using the Student's *t*-test. For physicians', patients' and clinical interviews discrete variables, *χ*^2^ tests were performed. Finally, to test our hypothesis, physicians' communication skills, the upper and lower quartiles of physicians in respect of their Rotter I-E scale scores, were compared using the Student's *t*-test.

## RESULTS

### Physicians' socioprofessional characteristics

Owing to the low response rate to the recruitment procedure (only 90 potentially interested subjects responded to the mail), 214 medical specialists dealing with cancer patients, including the 90 potentially interested, were actively contacted by phone. A total of 163 of them were met individually, and 21 information sessions were also organised in institutions devoted to cancer care. A total of 173 physicians were met during those sessions. Following this process, 113 physicians registered to the training, 81 physicians completed the simulated interview and 75 completed the clinical interview. Comparison of physicians who completed and physicians who did not complete the clinical interview showed no statistically significant differences for age, gender and number of years of practice. The reasons of the very small number of physicians willing to enter the training programme are the training and the assessment procedure durations that were often not compatible with their workload.

Among the 81 medical specialists who completed the simulated interview, 45 were male and 36 were female. Their mean age was 42 years old (s.d.=7.3). Our sample included 22 oncologists (27.2%), 9 radiotherapists (11.1%), 10 haematologists (12.4%), 18 gynaecologists (22.2%) and 22 other specialities (27.2%) (7 lung specialists (8.6%), 3 gastroenterologists (3.7%), 1 palliative care specialist (1.2%), 2 ENT specialists (2.5%), 2 geriatricians (2.5%), 2 general surgeons (2.5%), 1 plastic surgeon (1.2%), 1 dermatologist (1.2%), 1 medical biology specialist (1.2%) and 2 urologists (2.5%)). Only six of them have not achieved their medical specialisation training (7.4%). Physicians had in mean 16 years (s.d.=7.2) of medical practice and 14 years (s.d.=7.5) of practice in oncology. In average, they had cared 25 cancer patients (s.d.=22.5) in the last week. In all, 69 of them had hospital practice (85.2%), 39 practice in a 1-day clinic (48.2%) and 29 had private practice (35.8%). Finally, 41 physicians (50.6%) had some communication skills training in the last year (workshops, readings, conferences, etc.).

Mean physicians' score on the Rotter's I-E scale was 9.23 (s.d.=3.46). Our physicians' sample is thus more external than the family physicians' sample of Revicki and May's study (1985). In our statistic analysis, the upper and lower quartiles of the Rotter's I-E scale distribution were chosen to define interviews led by physicians with internal LOC (*n*=22) (Rotter I-E score from 0 to 7) or physicians with external LOC (*n*=18) (Rotter I-E higher than 11). Concerning their socioprofessional characteristics, no statistically significant differences were found between both groups except for the fact that all the haematologists were in the group of physicians with external LOC and the two physicians still in medical specialisation training were in the group of physicians with internal LOC (Table 1

**Table 1:** Socioprofessional characteristics of physicians with internal and external LOC (upper and lower quartiles of the Rotter I-E scale scores distribution)

	**Internal LOC**		**External LOC**	
*Age (years)*				
Mean (s.d.)	426	(6.3)	392	(5.7)
*Gender*				
Male	16	(72.7)	8	(44.4)
Female	6	(27.3)	10	(55.6)
*Medical speciality*				
Oncology	5	(22.7)	7	(38.9)
Radiotherapy	3	(13.6)	2	(11.1)
Haematology	—	—	4	(22.2)
Gynaecology	4	(18.2)	3	(16.7)
Others	10	(45.5)	2	(11.1)
*Medical specialisation training achieved*				
Yes	20	(90.9)	18	(100)
No	2	(9.1)	—	—
*Medical practice (years)*				
Mean (s.d.)	170	(6.5)	137	(5.5)
*Medical practice in oncology (years)*				
Mean (s.d.)	133	(6.8)	110	(6.4)
*Number of cancer patients cared during last week*				
Mean (s.d.)	187	(17.3)	262	(18.0)
*Medical practice*				
In hospital	18	(81.8)	15	(83.3)
In one-day clinic	10	(45.5)	8	(44.4)
Private	6	(27.3)	5	(27.8)
*Previous training in communication skills in the last year*				
Workshops, readings, conferences and others	10	(45.5)	9	(50.0)

Except when stated otherwise, values are expressed as frequencies, percentages are in brackets. No statistically significant differences were found between both groups except for the fact that all the haematologists were in the group of physicians with external LOC and the two physicians still in medical specialisation training were in the group of physicians with internal LOC.

).

### Physicians' attitudes on psychosocial aspects of cancer, burnout symptoms and job stress

Mean physicians' scores on SDAQ scale were 2.79 as regards attitudes towards oneself (s.d.=0.57), 3.10 for attitudes towards cancer and death (s.d.=0.66), 2.34 for attitudes linked to personal growth (s.d.=0.62), 2.55 for attitudes linked to professional relationships (s.d.=0.69), 2.86 for occupational attitudes (s.d.=0.62) and 2.74 for the total score (s.d.=0.49). As the neutral score on those scales is set at 4, our subjects' have relatively positive attitudes on psychosocial aspects of cancer. Student's *t*-test comparison of means showed no significant difference between physicians with internal and external LOC on those measures (Table 2

**Table 2:** Comparisons of attitudes on psychosocial aspects of cancer, burnout symptoms and job stress between physicians with internal and external LOC (upper and lower quartiles of the Rotter I-E scale scores distribution) (parametric Student's *t*-test)

	**Internal LOC**		**External LOC**			
	***M***	**s.d.**	***M***	**s.d.**	***t***	***P***
*Semantic differential attitude questionnaire (SDAQ)*						
Attitudes towards oneself	2.75	0.61	2.85	0.53	−0.541	0.591
Attitudes towards cancer and death	3.19	0.73	3.16	0.66	0.094	0.926
Personal growth	2.21	0.77	2.49	0.48	−1.315	0.197
Professional relationships	2.55	0.64	2.78	0.85	−0.994	0.327
Occupational attitudes	2.90	0.74	2.84	0.63	0.243	0.810
Total score (SDAQ)	2.74	0.54	2.83	0.52	−0.525	0.603
*Maslach burnout inventory (MBI)*						
Emotional exhaustion	17.82	8.24	20.00	6.14	−0.931	0.358
Personal growth	40.00	5.54	36.11	4.16	2.463	0.018
Depersonalisation	4.86	3.66	7.56	4.12	−2.189	0.035
*Job stress survey (JSS)*						
Severity	154.68	19.84	169.50	32.13	−1.789	0.082
Frequency	146.18	40.06	152.67	44.58	−0.484	0.631
Index (severity×frequency)	76.64	22.95	85.06	30.76	−0.991	0.328

).

Physicians' mean MBI scores were 18.68 for emotional exhaustion (s.d.=8.12), 6.26 for depersonalisation (s.d.=4.25) and 38.94 for personal growth (s.d.=5.07). As regards those three dimensions, our subjects presented thus a middle level of burnout (Dion and Réjean, 1994). The mean score in the personal growth scale in the group of physicians with internal (*M*=40.00; s.d.=5.54) was higher than in the group of physicians with external LOC (*M*=36.11; s.d.=4.16) (*t*=2.463; *P*=0.018). Moreover, mean score in the depersonalisation scale in the group of physicians with internal (*M*=4.86; s.d.=3.66) was lower than in the group of physicians with external LOC (*M*=7.56; s.d.=4.12) (*t*=-2.189; *P*=0.035) ( Table 2). Thus, for those dimensions, physicians with internal presented a lower level of burnout than physicians with external LOC (Dion and Réjean, 1994).

Finally, physicians' mean scores on the JSS were 159.19 for the severity (s.d.=26.61), 139.27 for the frequency (s.d.=40.09) and 74.17 for the overall job stress index (s.d.=26.26). Our subjects' perceived stress severity, frequency and job stress index scores were thus only slightly above the median level. Student's *t*-test comparison of means showed no statistically significant difference between physicians with internal and external LOC even though those latter tended to express more perceived stress severity (*M*=169.50; s.d.=32.13) than physicians with internal LOC (*M*=154.68; s.d.=19.84) (*t*=−1.789; *P*=0.082) ( Table 2).

### Patients' and clinical interviews' characteristics

Among the 75 patients met by physicians in the clinical interview, 30 were male and 45 were female. Their mean age was 58 years old (s.d.=13.5). A total of 27 of them had achieved junior high school or less (36%), 19 were high school graduate (25.3%) and 29 were college or university graduates (38.7%); 60 of them were able to achieve their daily life activities (80%) (KPS score from 80 to 100).

Patients' mean HADS total emotional distress score was 11.6 (s.d.=6.3). In average, patients thus did not show any emotional distress disorder. Their mean MHLC scores were 23.9 (s.d.=5.3) for the internal health locus of control subscale, 21.2 (s.d.=6.5) for the external chance health locus of control subscale and 25.2 (s.d.=6.5) for the external powerful others health locus of control subscale.

In all, 64 patients had a solid tumour diagnosis (85.3%) and 55 a prognosis of 1 year or more (73.3%). In mean, the diagnosis has been established 29.9 months before the interview (s.d.=41.6). For 21 patients, the tumour was in progression (28%) and 43 of them were currently following a cancer treatment (57.3%). Information given to the patient was diagnosis related in 32 interviews (42.7%). In total, 26 patients were given neutral news (34.7%), 22 good news (29.3%) and 27 bad news (36.0%).

Comparisons of interviews' characteristics led by physicians with internal and external LOC showed no statistically significant differences (Table 3

**Table 3:** Characteristics of interviews led by physicians with internal and external LOC (upper and lower quartiles of the Rotter I-E scale scores distribution)

	**Internal LOC**		**External LOC**	
*Age (years)*				
Mean (s.d.)	548	(15.5)	576	(13.1)
*Gender*				
Male	9	(45.0)	4	(23.5)
Female	11	(55.0)	13	(76.5)
*School level completed*^a^				
Junior high school or less	6	(30.0)	3	(17.7)
High school graduate	6	(30.0)	6	(32.3)
College or university graduation	8	(40.0)	8	(47.1)
*Karnofsky performance status (KPS)*^a^				
80 or more	14	(70.0)	14	(82.4)
Less than 80	6	(30.0)	3	(17.7)
*Hospital anxiety and depression scale (HADS)*				
Emotional distress total mean scores (s.d.)	109.5	(52.8)	118.2	(62.4)
*Multidimensional health locus of control (MHLC)*				
Internal HLC mean scores (s.d.)	246.0	(52.2)	226.5	(54.5)
External Chance HLC mean scores (s.d.)	209.0	(72.8)	218.2	(56.7)
External Powerful Others HLC mean scores (s.d.)	258.0	(68.6)	258.2	(72.1)
*Type of cancer*^a^				
Solid tumour	19	(95.0)	14	(82.4)
Haematologic cancer	1	(5.0)	3	(17.7)
*Prognosis*				
Less than 1 year	7	(35.0)	4	(23.5)
1 year or more	13	(65.0)	13	(76.5)
*Disease status*				
In remission, no change or too early to assess	13	(35.0)	12	(70.6)
In progression	7	(65.0)	5	(29.4)
*Current cancer treatment*				
Yes	9	(45.0)	11	(64.7)
No	11	(55.0)	6	(35.3)
*Months since diagnosis*				
Mean (s.d.)	287	(43.1)	365	(54.7)
*Type of information*				
Diagnosis related	11	(55.0)	5	(29.41)
Not diagnosis related	9	(45.0)	12	(70.59)
*Type of news*				
Neutral	6	(30.0)	8	(47.1)
Good	5	(25.0)	5	(29.4)
Bad	9	(45.0)	4	(23.5)

Except when stated otherwise, values are expressed as frequencies, percentages are in brackets. No statistically significant differences were found between both groups.

a *χ*^2^ not applicable because of a lack of observations in the cells.

).

### Physicians' LOC and communication skills

Table 4

**Table 4:** Comparisons of communicative functions used by physicians with internal and external LOC in the simulated interview and the clinical interview (upper and lower quartiles of the Rotter I-E scale scores distribution) (Parametric Student's *t*-test)

	**Simulated interview**						**Clinical interview**					
	**Internal LOC**		**External LOC**				**Internal LOC**		**External LOC**			
	***M***	**s.d.**	***M***	**s.d.**	***t***	***P***	***M***	**s.d.**	***M***	**s.d.**	***t***	***P***
Introducing–closing	2.5	1.7	2.5	1.4	−0.037	0.971	2.4	1.8	3.6	3.3	−1.346	0.191
Assessing, checking, summarising	32.0	12.9	31.5	10.2	0.128	0.899	19.6	12.2	25.5	10.3	−1.571	0.125
Acknowledging	21.6	9.2	22.1	9.4	−0.167	0.868	25.5	14.0	24.9	8.2	0.153	0.879
Appropriate information	5.9	4.4	9.8	4.9	−2.658	0.011	27.5	13.9	26.7	10.2	0.192	0.849
Premature information	24.6	10.1	22.1	9.3	0.805	0.426	12.7	8.8	6.4	5.4	2.550	0.015
Reassuring, empathy	1.2	1.4	1.8	2.8	−0.931	0.357	0.3	0.5	0.5	1.3	−0.840	0.407
Premature reassurance	4.0	3.6	2.8	1.5	1.439	0.161	0.6	0.7	0.2	0.6	1.773	0.085
Educated guesses, confronting, alerting to reality	2.1	2.6	1.7	1.9	0.525	0.603	0.0	0.2	0.2	0.3	−1.416	0.169
Negotiating	1.3	1.6	1.4	2.6	−0.189	0.851	1.0	1.0	0.9	1.2	0.162	0.872
Unrated function	4.8	4.4	4.2	3.9	0.435	0.666	10.5	8.6	11.1	8.5	−0.209	0.836

shows results of parametric Student's *t*-test comparison of mean frequencies of functions used by physicians with internal and external LOC as rated with the CRCWEM. As one can see, in the simulated interview, only the mean frequency of the appropriate information giving function was significantly different and higher in the group of physicians with external (*M*=9.8; s.d.=4.9) than in the group of physicians with internal LOC (*M*=5.9; s.d.=4.4) (*t*=−2.658; *P*=0.011). In the clinical interview, the mean frequency of the premature information giving function was significantly different and lower in the group of physicians with external (*M*=6.4; s.d.=5.4) than in the group of physicians with internal LOC (*M*=12.7; s.d.=8.8) (*t*=2.550; *P*=0.015).

## DISCUSSION

This paper explores the relations between medical specialists' LOC and communication skills established in oncological interviews. Our purpose was to assess whether significant differences exist in communication skills used by physicians with a more internal or external LOC orientation. We hypothesised that physicians with external LOC, as they believe that life outcomes are controlled by external forces such as other individuals, would be more interested in others' concerns than physicians with internal LOC and would consequently use more appropriate assessment, informative and supportive skills with cancer patients.

Before drawing any conclusions, it must be kept in mind that our sample is more external than the American family physicians sample of Revicki and May (1985). Even if cultural differences could not be excluded, medical practice in oncology may explain this higher level of externality in our sample of physicians. Indeed, facing the uncertainty inherent to cancer and its treatment could increase physicians' beliefs that life outcomes are in part controlled by external factors like chance, fate or luck. Moreover, it could not be excluded that more internal oncologists would be more reluctant or would feel less need to take part in communication skills training as they may feel in control of their relationships with their cancer patients.

As far as socioprofessional characteristics of both groups are concerned, it should be noted, first of all, that all haematologists were in the group of physicians with external LOC. Researches may be needed to study factors specific to this medical practice that could lead haematologists to have a more external LOC. Moreover, it could not be excluded that among medical specialists, physicians with external LOC have personal characteristics that could lead them to choose to work in this medical speciality. Secondly, the fact that the two physicians who were still in medical specialisation training were in the group of physicians with internal LOC may be linked with the fact that education programmes may increase the internality of the participants (see for example Dubois, 1988). Finally, preliminary Student's *t*-test comparisons between both groups of physicians showed that physicians with external LOC reported less personal growth and a higher level of depersonalisation on the MBI subscale than physicians with internal LOC. Physicians with external LOC, moreover, tended to report more perceived stress severity than physicians with internal LOC. This confirms results of numerous previous researches that have established that subjects with external LOC are less efficient in coping with stress (see for example Krause and Stryker, 1984).

As far as communication skills are concerned, for a same level of assessment and supportive functions, physicians with external LOC gave more appropriate information in the highly emotional simulated interview and less premature information in the clinical interview than physicians with internal LOC. Our results thus confirm that physicians' LOC can influence their communication style. Indeed, if our results do not confirm any difference between physicians with internal and external LOC in the assessment and supportive skills used, they indicate that physicians with an external LOC are more efficient in the way they give information.

It is important to note that in the CRCWEM, information giving is rated as appropriate only if it is grown after exploring patients' feelings and if the information given is realistic and takes into account the interview's coherence. On the contrary, if information is given without taking into account those criteria, it is rated as premature information. Our result indicates that physicians with external differ from physicians with internal LOC in their respect of those criteria when they inform the patient. Physicians with external compared with physicians with internal LOC thus either take the results of their assessment more into account when they inform their patients or assess more often the patients' concerns before giving the information.

This result indicates that physicians with internal and physicians with external LOC may be different in their abilities to inform cancer patients appropriately in a high as well as in a low emotional level. It represents thus a first empirical evidence that physicians can have different communication styles when they inform cancer patients. Ideally, this communication style has to be adapted to the patients' and interviews' characteristics. However, it seems that the physicians' LOC is more influent on those styles than patients' or interviews' characteristics.

One limitation of this study has to be underlined. The use of effective training techniques as well as valid communication skills assessment procedures is time consuming. Physicians who accepted to participate in the training programme were thus the most motivated. As a result, the representativeness of our sample is limited to medical specialists who are motivated to improve their communication skills with cancer patients and who are the most convinced of the importance of training. We can hypothesise however that differences would be all the more important among physicians less conscious of the importance of using effective communications skills with their patients.

The results of this study can have implications for training programmes in communication skills aimed for medical specialists caring cancer patients. Indeed, as giving appropriate information to the patient seems to be determined by physician's LOC, one objective of training programmes could be to increase the ability of physicians with external LOC to inform cancer patients by taking more into account patients' or interviews' characteristics. Moreover, only active and participative methods of training seem to be effective (including constructive exchanges and observations between participants; for a review, see Roter and Fallowfield, 1998). Thus, communication skills training programme that include physicians with internal and external LOC in constructive interactions may be useful for the former to reach this objective.
