# Impact of medical specialists' locus of control on communication skills in oncological interviews

**DOI:** 10.1038/sj.bjc.6600966

**Published:** 2003-06-10

**Authors:** Y Libert, P Janne, D Razavi, I Merckaert, P Scalliet, N Delvaux, A-M Etienne, S Conradt, J Klastersky, J Boniver, Ch Reynaert

**Affiliations:** 1 Universite Catholique de Louvain, Belgium; 2 Universite Libre de Bruxelles, Belgium; 3 Universite de Liege, Belgium

**Correction to:** *British Journal of Cancer* (2003) **88**, 502–509. doi:10.1038/sj.bjc.6600797

Unfortunately because of a typesetting error, Tables 1

**Table 1:** Socioprofessional characteristics of physicians with internal and external LOC (upper and lower quartiles of the Rotter I-E scale scores distribution)

	**Internal LOC**		**External LOC**	
*Age (years)*				
Mean (s.d.)	43	(6.3)	39	(5.7)
*Gender*				
Male	16	(72.7)	8	(44.4)
Female	6	(27.3)	10	(55.6)
*Medical speciality*				
Oncology	5	(22.7)	7	(38.9)
Radiotherapy	3	(13.6)	2	(11.1)
Haematology	—	—	4	(22.2)
Gynaecology	4	(18.2)	3	(16.7)
Others	10	(45.5)	2	(11.1)
*Medical specialisation training achieved*				
Yes	20	(90.9)	18	(100)
No	2	(9.1)	—	—
*Medical practice (years)*				
Mean (s.d.)	17	(6.5)	14	(5.5)
*Medical practice in oncology (years)*				
Mean (s.d.)	13	(6.8)	11	(6.4)
*Number of cancer patients cared during last week*				
Mean (s.d.)	19	(17.3)	26	(18.0)
*Medical practice*				
In hospital	18	(81.8)	15	(83.3)
In one-day clinic	10	(45.5)	8	(44.4)
Private	6	(27.3)	5	(27.8)
*Previous training in communication skills in the last year*				
Workshops, readings, conferences and others	10	(45.5)	9	(50.0)

Except when stated otherwise, values are expressed as frequencies, percentages are between brackets. No statistically significant differences were found between both groups except for the fact that all the haematologists were in the group of physicians with external LOC and the two physicians still in medical specialisation training were in the group of physicians with internal LOC.

and 3

**Table 3:** Characteristics of interviews led by physicians with internal and external LOC (upper and lower quartiles of the Rotter I-E scale scores distribution)

	Internal LOC		**External LOC**	
*Age (years)*				
Mean (s.d.)	55	(15.5)	58	(13.1)
*Gender*				
Male	9	(45.0)	4	(23.5)
Female	11	(55.0)	13	(76.5)
*School level completed*^a^				
Junior high school or less	6	(30.0)	3	(17.7)
High school graduate	6	(30.0)	6	(32.3)
College or university graduation	8	(40.0)	8	(47.1)
*Karnofsky performance status (KPS)*^a^				
80 or more	14	(70.0)	14	(82.4)
Less than 80	6	(30.0)	3	(17.7)
*Hospital anxiety and depression scale (HADS)*				
Emotional distress total mean scores (s.d.)	11.0	(5.3)	11.8	(6.2)
*Multidimensional health locus of control (MHLC)*				
Internal HLC mean scores (s.d.)	24.6	(5.2)	22.7	(5.5)
External Chance HLC mean scores (s.d.)	20.9	(7.3)	21.8	(5.7)
External Powerful Others HLC mean scores (s.d.)	25.8	(6.9)	25.8	(7.2)
*Type of cancer*^a^				
Solid tumour	19	(95.0)	14	(82.4)
Haematologic cancer	1	(5.0)	3	(17.7)
*Prognosis*				
Less than 1 year	7	(35.0)	4	(23.5)
1 year or more	13	(65.0)	13	(76.5)
*Disease status*				
In remission, no change or too early to assess	13	(35.0)	12	(70.6)
In progression	7	(65.0)	5	(29.4)
*Current cancer treatment*				
Yes	9	(45.0)	11	(64.7)
No	11	(55.0)	6	(35.3)
*Months since diagnosis*				
Mean (s.d.)	29	(43.1)	37	(54.7)
*Type of information*				
Diagnosis related	11	(55.0)	5	(29.4)
Not diagnosis related	9	(45.0)	12	(70.6)
*Type of news*				
Neutral	6	(30.0)	8	(47.1)
Good	5	(25.0)	5	(29.4)
Bad	9	(45.0)	4	(23.5)

Except when stated otherwise, values are expressed as frequencies, percentages are in brackets. No statistically significant differences were found between both groups.

a *χ*^2^ not applicable because of a lack of observations in the cells.

were reproduced incorrectly. The correct versions are printed below:

The publisher would like to apologise for any inconvenience this may have caused.

